# Mesoporous Silica Skin on Clay Nanotubes for Carbon Capture

**DOI:** 10.1021/acsanm.5c01071

**Published:** 2025-06-17

**Authors:** Borui Wang, Oluwole Ajumobi, Jibao He, Julia A. Valla, Vijay T. John

**Affiliations:** † Department of Chemical & Biomolecular Engineering, 5783 Tulane University, 6823 St. Charles Avenue, New Orleans, Louisiana 70118, United States; ‡ Coordinated Instrumentation Facility, 5783 Tulane University, 6823 St. Charles Avenue, New Orleans, Louisiana 70118, United States; § Department of Chemical & Biomolecular Engineering, University of Connecticut, Storrs, Connecticut 06269, United States

**Keywords:** halloysite nanotubes, MCM-41, mesoporous materials, polyethylenimine, carbon capture, hierarchical porosity

## Abstract

Amine-based adsorbents are considered extremely promising candidates for their efficacy in CO_2_ capture. In this study, we explore the enhancement of CO_2_ adsorption capacity through the development of a hierarchically porous material containing a mesoporous silica coating on halloysite nanotubes (HNTs), a naturally occurring clay material. The generation of a mesoporous MCM-41 skin on HNTs increases the surface area from about 60 to 400 m^2^/g while maintaining structural integrity. This significant increase in the surface area helps enhance amine loading. The synthesis of the MCM-41/HNT (MHNT) composite particles was achieved via an aerosol-assisted method, allowing rapid coating formation of a spindle-shaped skin on the HNT external surface and leading to a hierarchical porosity that supports both large pores in the HNT lumen and small pores in the MCM-41 coating. Poly­(ethylenimine) (PEI)-loaded MHNT adsorbents exhibit superior CO_2_ adsorption capacities compared to adsorbents of PEI loaded into pristine HNT, with a 27% increase in the adsorption capacity. This work underscores the effectiveness of mesoporous skin in increasing amine adsorption efficiency on clay-based adsorbents, providing a pathway for the development of high-capacity, durable materials in carbon capture technologies.

## Graphical abstract

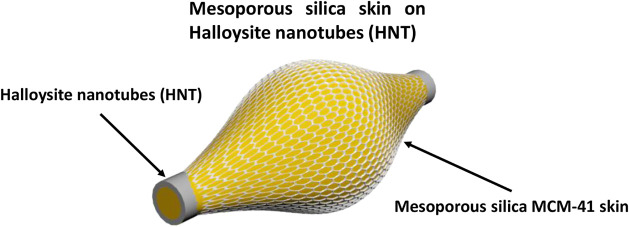

## 1 Introduction

Amine adsorbents for CO_2_ capture are a prominent class of materials used in the mitigation of carbon dioxide emissions from industrial and power generation sources.^1–3^ These adsorbents typically leverage the chemical reactivity of amine groups to selectively bind CO_2_ molecules, making them highly effective for carbon capture applications. The fundamental mechanism involves the formation of a carbamate or bicarbonate when the amine reacts with CO_2_ (eqs 1 and 2),^4–6^ a reversible process that allows for the subsequent release and capture of CO_2_ in a controlled manner.^7^

1 $${\mathrm{RNH}}_{2}+{\mathrm{CO}}_{2}↔{{\mathrm{RNH}}_{2}}^{+}{\mathrm{COO}}^{-}$$

2 $${{\mathrm{RNH}}_{2}}^{+}{\mathrm{COO}}^{-}+{\mathrm{RNH}}_{2}↔{\mathrm{RNHCOO}}^{-}+{{\mathrm{RNH}}_{3}}^{+}$$

The appeal of amine-based adsorbents lies in their high efficiency and selectivity for CO_2_, as well as their adaptability to the existing infrastructure. These materials can be synthesized in various forms, including being impregnated or grafted onto solid supports such as silica,^8^ alumina,^9^ or polymers,^10^ which enhances their stability and usability.^11^ Although solid amine-based adsorbents have effectively removed CO_2_ from flue gas and the atmosphere, the current requirements call for improved performance. Consequently, there is an urgent need to develop adsorbents with a higher amine density to enhance the CO_2_ adsorption capacity.

Halloysite nanotubes (HNTs) are aluminosilicate clay materials with a hollow structure.^12,13^ They are not only inexpensive but also environmentally friendly and readily accessible in large quantities.^14,15^ Their aluminosilicate composition (Al_2_Si_2_O_5_(OH)_4_), which is chemically similar to kaolinite, imparts good chemical stability.^16^ These nanotubes have a positively charged inner surface and a negatively charged external surface.^14,15,17–20^ HNTs have a length of 0.5–3 μm and a lumen diameter of 20–30 nm.^21,22^ The lumen functions as a large mesopore capable of hosting guest molecules, allowing infiltration and subsequent packing of species such as polyethylenimine (PEI) for CO_2_ capture studies.^23–26^ However, HNTs intrinsically do not have a high surface area, with a surface area of approximately 47 m^2^/g, which leads to a relatively low loading of PEI through adsorption.^14^ Further addition of PEI leads to the formation of an external thick coating and a diffusional barrier for CO_2_ leading to a decrease in capture.^27^

Our concept involves the generation of a mesoporous silica skin on the surface of HNTs in an attempt to significantly increase the surface area of the material and thereby develop new application potential for HNTs. Traditionally, the surface area of HNTs is modified by controlled etching of the silica–aluminate wall of the nanotube.^28–30^ This leads to improvements in the surface area from 47 m^2^/g to about 80–300 m^2^/g but has the drawback of weakening the wall structure.^31,32^

Our approach for generating a mesoporous skin on the external surface is hypothesized to lead to a much larger surface area without compromising the mechanical strength of the HNT wall. We therefore synthesize mesoporous MCM-41 on the external surface of HNTs in a one-step aerosol-assisted process (Figure 1).^22,33,34^ MCM-41 is characterized by a highly organized hexagonal pore structure, with a pore diameter that varies between 2 and 4 nm, and it exhibits an extremely high surface area of up to 1500 m^2^/g.^35^ In this procedure, the precursor solution containing the templating surfactant cetyltrimethylammonium bromide (CTAB), tetraethoxysilane (TEOS), and HNTs is mixed in an ethanol–water solution, then aerosolized and directed into a heated tube furnace.

**Figure 1:**
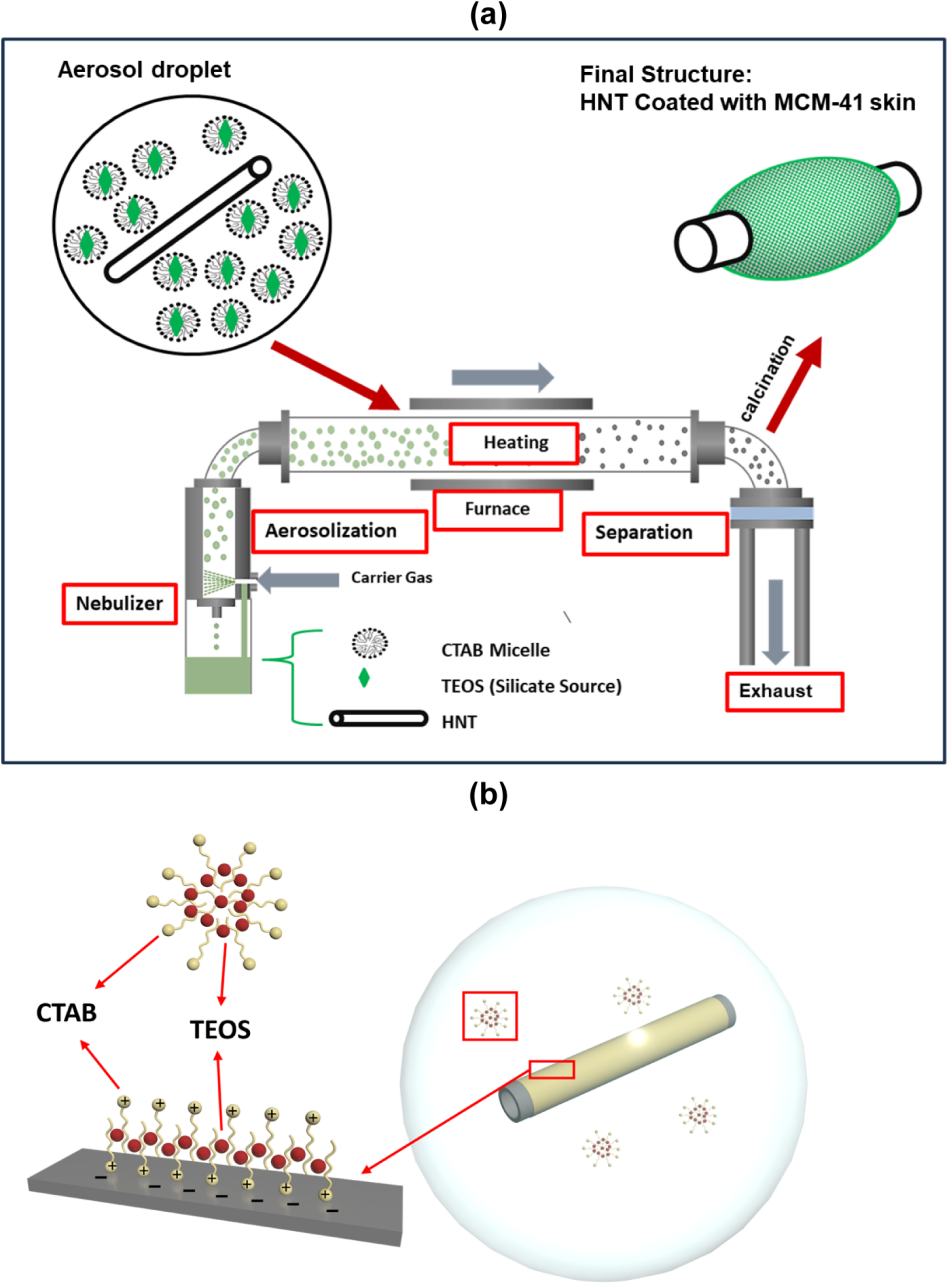
(a) Schematic illustration of the aerosol-assisted synthesis technique. Reproduced from Ajumobi and coworkers, Energy & Fuels 2023, 37 (16), 12079–12088. https://doi.org/10.1021/acs.energyfuels.3c01318. Copyright 2023 American Chemical Society. (b) Schematic illustration of an aerosol droplet containing halloysite nanotubes with CTAB molecules adsorbed on its surface.

In generating a mesoporous skin on the HNT, we hypothesize that adsorption of the cationic CTAB on the anionic external surface of the HNT will allow nucleation and growth of MCM-41 on the external surface of the HNT as the droplet evaporates. This is a rough equivalent of the evaporation-induced self-assembly process of forming mesoporous silica films on surfaces, as pioneered by Lu and coworkers.^36^ Our approach is distinguished by its focus on rapidly forming coatings on halloysite nanotubes. The intrinsic scalability of the aerosol technique will, therefore, allow the generation of large amounts of such coated HNTs. There is thus the possibility of new applications where both the HNT lumen (with small surface areas but large pores) and the mesoporous coatings (with small pores but large surface areas) can be synergistically used. The current objective is to synthesize materials that have a hybrid hierarchical structure, consisting of small mesopores (∼3 nm) from MCM-41 and larger mesopores (∼20 nm) from HNTs, and to examine their potential for CO_2_ capture with embedded amines in the pores.

In earlier work from this laboratory, we used the aerosol method to insert HNTs into large particles of MCM-41 to serve as nanostraws and provide diffusional pathways for species entry and egress from the interior of MCM-41.^37–39^ We showed that this nanostraw-based morphology had implications for both CO_2_ capture and reaction- and diffusion-based processes. This paper describes a complementary approach where the morphology of the HNT is largely kept intact, and a skin of MCM-41 is formed to greatly enhance the surface area of the HNT.

## 2 Experimental Section

### 2.1 Materials

Tetraethoxysilane (TEOS, 98%), cetyltrimethylammonium bromide (CTAB, 95%), and hydrochloric acid (HCl, 37%) were purchased from Sigma-Aldrich. These chemicals were used without any modifications. Branched PEI (600 Mw) was purchased from Thermo Scientific. HNTs (Guangzhou Runwo, China) were obtained as a gift from Yuri Lvov (Institute for Micromanufacturing, Louisiana Tech University). Deionized (DI) water with a resistance of 18.2 MΩ was obtained from an Elga water purification system (Medica DV25).

### 2.2 Synthesis of the Composite Particles of MCM-41 Skin on Halloysite Nanotubes

MCM-41/HNT (MHNT) composite particles were synthesized using an aerosol-assisted method, which was slightly modified from previously reported techniques.^22^ Initially, 0.084 g of CTAB was dissolved in 80 mL of ethanol, and 0.1 g of HNT particles were added to the CTAB solution, followed by stirring for 20 min. 200 μL of TEOS was then gradually added to the mixture while being continuously stirred magnetically. Following this, 20 mL of 0.1 M HCl was incorporated, and the mixture was stirred at ambient temperature for 15 min. The precursor solution was subsequently added to a nebulizer (Micro Mist, Teleflex, MMAD: 2.1 μm).^40^ Here, the solution was aerosolized into fine droplets under nitrogen gas flow at 2.5 L/min. These droplets were then conveyed into a heating chamber, which consisted of a tube furnace (76 cm in length and 5 cm in diameter) for rapid condensation of TEOS into silica at 440 °C. At the furnace’s end, a filter paper (Merck Millipore Ltd., with a pore size of 0.22 μm) collected the dried particles. Heating coils were used to raise the filtration setup temperature to 80 °C, preventing moisture condensation. The collected particles were subjected to calcination in air at 550 °C for 8 h, with a heating rate of 5 °C/min, to burn and eliminate CTAB. To better compare HNTs with MHNTs, HNTs were also calcined under the same conditions.

### 2.3 Loading of the HNT and MHNT Composites with PEI

The incorporation of polyethylenimine (PEI) into the bare HNT and MHNT was conducted using the wet impregnation technique and vacuum suction.^41^ To achieve a 30 wt % PEI loading in the HNT or MHNT, 0.05 g of PEI was mixed with 5 mL of absolute ethanol and stirred for 10 min to ensure complete dissolution. Subsequently, 0.15 g of HNT or MHNT were added to this PEI solution. The mixture was subjected to ultrasonic sonication for 10 min and then magnetically stirred for 24 h at ambient temperature. This mixture was then placed in a 25 mL round-bottomed flask and connected to a rotary evaporator set at 60 °C. A vacuum of 100 mbar was applied to facilitate the infusion of PEI into the HNT channels while also aiding in the evaporation of the ethanol. The sample was then left to dry under a reduced vacuum of 5 mbar for an hour. The dried PEI-loaded HNT and MHNT samples were collected and stored in a sealed container. Adsorbents with PEI loadings ranging from 20 to 50 wt % were also synthesized using an identical procedure, adjusting the PEI-to-solid particle mass ratio for MHNT particles.

### 2.4 Material Characterization

Structural characterization of MHNT samples was performed by using powder X-ray diffraction (XRD) analysis. This was conducted with a Rigaku Miniflex II instrument employing Cu Kα radiation at a wavelength of 1.54 Å, across a wide-angle scan range of 2θ from 5° to 70°. To evaluate the surface area and pore volume of the samples, sorption analysis was conducted using a Micromeritics ASAP 2020 instrument, based on the Brunauer–Emmett–Teller (BET) isotherm and Barrett–Joyner–Halenda (BJH) desorption cumulative method. For morphological analysis, scanning electron microscopy (SEM) and transmission electron microscopy (TEM) techniques were employed. SEM imaging was performed on a Hitachi S-4800 field emission SEM operating at 3 kV, with samples coated in a thin carbon layer using a Cressington Carbon Coater 208carbon via thermal evaporation to enhance conductivity and image quality. TEM images were captured using an FEI Tecnai G2 F30 twin TEM operating at 300 kV. The quantification of PEI on each sample was determined through weight loss analysis via thermogravimetric analysis (TGA) on a TA Instruments Q500 thermogravimetric analyzer over a temperature range from 25 to 710 °C. Furthermore, Fourier transform infrared (FT-IR) spectroscopy was used to identify the presence of functional groups on the PEI-loaded samples. The FTIR spectra were recorded with 32 accumulated scans at a resolution of 4 cm^–1^.

### 2.5 CO_2_ Capture Analysis

The CO_2_ capturing capabilities of HNT and MHNT samples with PEI were tested under dry conditions using TGA. For these tests, 10 mg of sample was placed on a platinum pan inside the TGA device. The procedure began by heating the sample at a rate of 5 °C per minute until reaching 105 °C, where it was held for 1 h under a flow of nitrogen gas to remove any pre-absorbed moisture or other gases. After this drying step, the temperature was lowered to 45 °C, and dry CO_2_ gas was introduced at a flow rate of 90 mL/min for 2 h to allow for full CO_2_ adsorption. The quantity of CO_2_ captured was assessed by measuring the weight change of the sample before and after exposure to CO_2_. This procedure was consistently repeated for all samples at an increased adsorption temperature of 75 °C, while the rest of the conditions were kept unchanged.

## 3 Results and Discussion

### 3.1 Material Characterization

The morphology of bare HNTs is shown in Figure 2. Figure 2a,b shows the typical long rod-like structure of HNTs with smooth surfaces. HNTs typically have an average length ranging from 0.5 to 3 μm, an outer diameter of approximately 60–80 nm, and an inner lumen of 15–30 nm^31,42^ (Figure 2c,d). Figure 3a,b shows the SEM images of the control sample of MCM-41. These images reveal spherical particles that exhibit a wide size distribution, ranging from approximately 50 nm to 2 μm. The SEM image of MCM-41 particles highlights their spherical morphology as obtained through the aerosol-assisted process. The TEM image in Figure 3c offers a detailed view of these MCM-41 particles, while the ordered array of pores is clearly visible in the high-resolution TEM image (Figure 3d).

**Figure 2:**
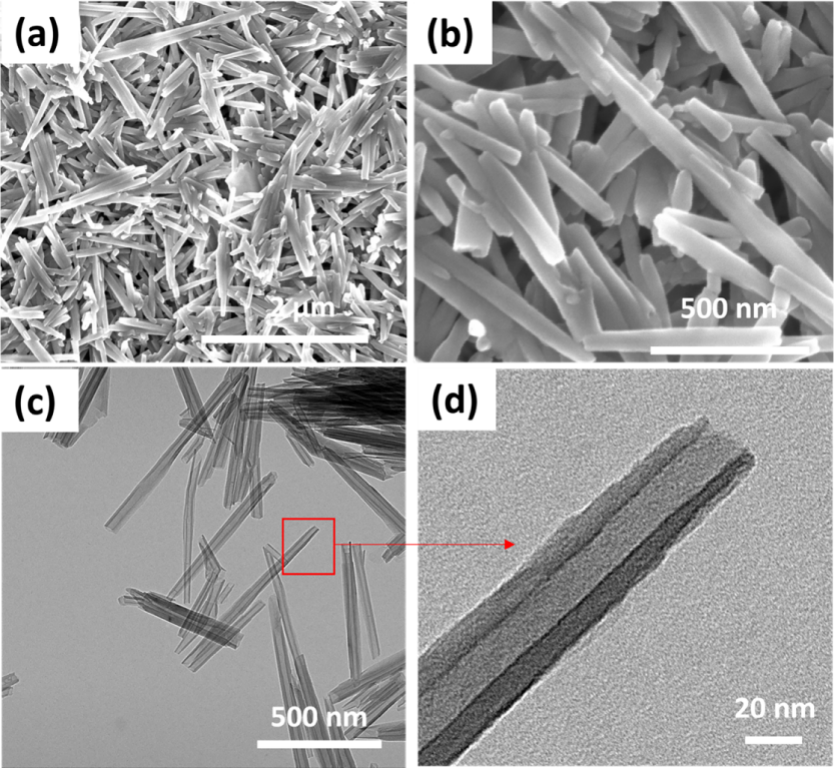
SEM image of (a) HNT and (b) high-resolution SEM image of the HNT. TEM image of (c) HNT and (d) high-resolution TEM image of HNT showing the hollow lumen of the HNT.

**Figure 3:**
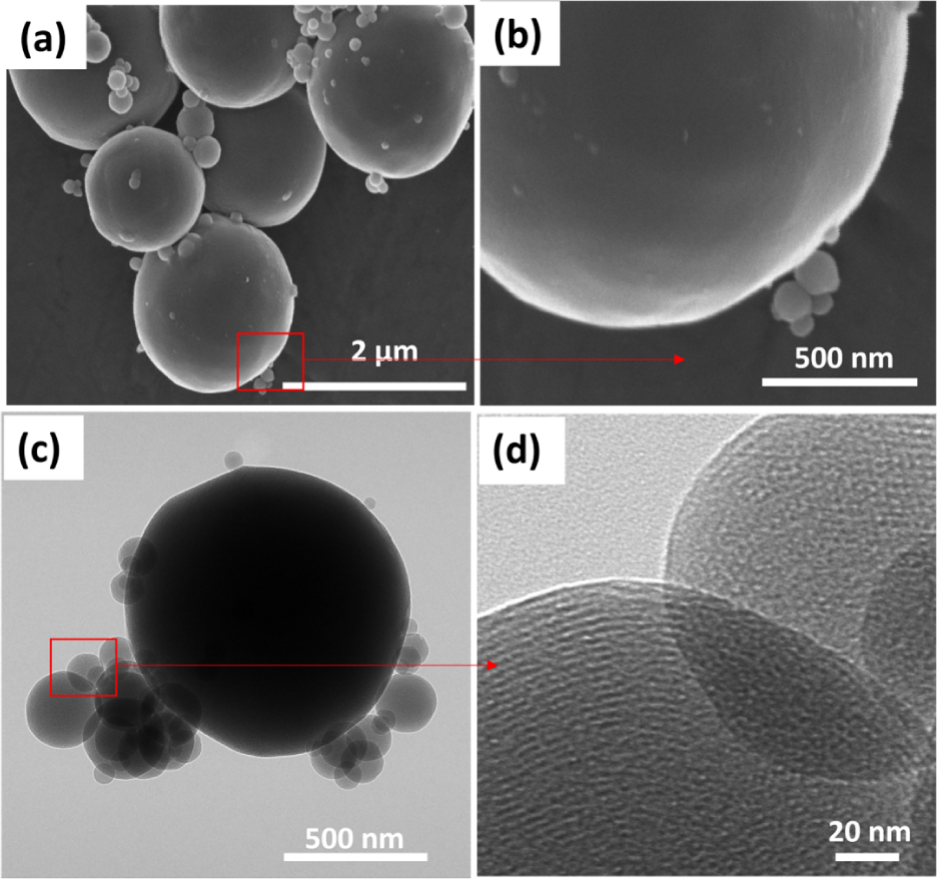
SEM image of (a) MCM-41 and (b) high-resolution SEM image of MCM-41. TEM image of (c) MCM-41 and (d) high-resolution TEM image of MCM-41 showing the presence of a hexagonal arrangement of mesopores.

Composite MHNTs were synthesized using the same method as for MCM-41, incorporating 65 wt % HNT particles into the MCM-41 precursor solution. In these experiments, the concentrations of HNT, TEOS, and CTAB were maintained at ∼1.0  g/L, 0.009 mol/L, and 0.0023 mol/L, respectively. These relatively diluted concentrations were chosen to suppress homogeneous nucleation and facilitate the controlled growth of mesoporous silica (MCM-41) skin on the HNT surface. Due to electrostatic interactions, the positively charged CTAB molecules adsorb onto the negatively charged silica walls of HNTs,^43^ creating a stable template for the organization of TEOS. Similar to generating mesoporous silica films on silica substrates, the process is guided by the interaction of surfactant-silica species with the surface,^44,45^ where controlled templating and orientation play critical roles in determining the final morphology and uniformity of the coating. The self-assembly of CTAB micelles acts as a structural template for the formation of mesoporous silica. After condensation of the silica source, MCM-41, with its distinct ordered mesoporous structure, is expected to form on the surface of the HNTs. In the aerosol-assisted method, this composition produces droplets in the 1–5  μm diameter range (MMAD ≈ 2.1 μm).^40^ Based on geometric estimates, a 2.1 μm droplet contains approximately 1.02 HNT particles on average. This stoichiometry ensures that most droplets contain a single HNT template, minimizing aggregation and favoring individual coating. However, due to stochastic variations, some larger droplets (∼5 μm) may contain multiple HNTs, while smaller ones may entirely lack HNTs.

SEM and TEM images reveal the resulting composite particle morphology. In the SEM image of MHNT (Figure 4a,b), we observe the formation of a novel spindle-shaped coating on the HNT with convex curvature. Our original hypothesis was that there would be a uniform coating of MCM-41 on each HNT. However, upon observing the unusual spindle-shaped morphologies of MCM-41 over the HNT, as shown in Figure 4, we realized that the formation mechanism was governed by solvent evaporation within the droplet. The receding areal coverage of the droplet leads to the roughly convex spindle shape of the MCM-41, where the thick part is due to the buildup of MCM-41 with TEOS still in the last stages of droplet evaporation. Additional small spherical satellite particles were also observed on the surface of the convex-shaped coatings on HNTs, which may have formed from smaller aerosol droplets lacking HNT templates. TEM imaging confirms these surface features and also indicates that multiple HNTs may be integrated into the spindle-shaped structures (Figure 4c). Figure 4d provides a high-resolution TEM image that illustrates the microporosity of the spindle-shaped shell and also indicates the mesoporous structures of the satellite particles. This thin shell introduces a high density of ∼3 nm mesopores, which increases the surface area while preserving accessibility. Based on the observed morphology, we suspect that such a structure may promote a more uniform distribution of PEI in the MHNT composite, help minimize diffusion limitations due to the thinness of the MCM-41 skin, and enhance CO_2_ capture performance.

**Figure 4:**
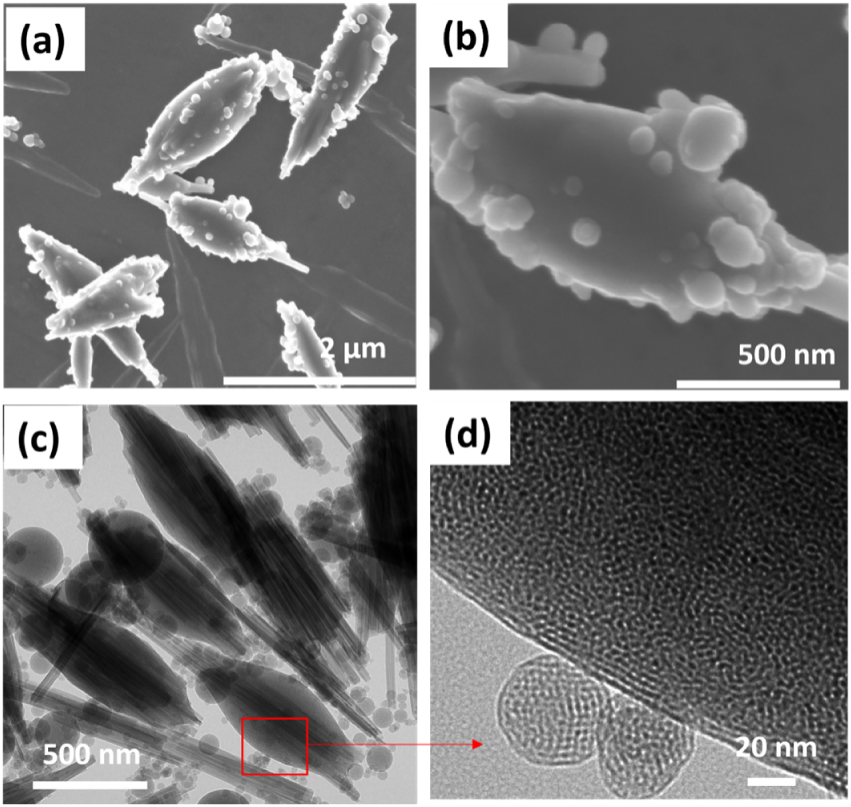
SEM image of (a) MHNT and (b) high-resolution SEM image of MHNT. TEM image of (c) MHNT and (d) high-resolution TEM image of MHNT showing the presence of the hexagonal arrangement of mesopores from MCM-41 skin.

We term the spindle-shaped coating on the HNT as a mesoporous “skin” that is formed over the HNT. Such structures seem unique to the aerosol process and can be explained as follows, with a schematic representation provided in Figure 5. As the droplets containing the precursor solution pass through the heated zone, rapid evaporation of the solvent occurs. During this evaporation process, the tubular structure of HNTs within the droplet provides a confined environment for the synthesis of MCM-41. This confinement effect ensures that the growth of the silica framework occurs along the length of the nanotubes. The restricted space around the HNT directs the formation of the mesoporous structure into an elongated shape. This effect is further enhanced by the uniform linear structure of the HNTs and the electrostatic interaction between CTAB and the external surface of the HNT. As a result, the MCM-41 skin gradually forms on the external surface of the HNT. Due to the contact between the external surface of the droplet and the heated gas, the solvent evaporates within the droplet. The balance between solvent evaporation and surface tension forces of the droplet can lead to the formation of spindle-like shapes. The shrinking droplet size, combined with the surface tension acting on the silica precursor and CTAB template, drives the formation of elongated, spindle-like structures on HNT.

**Figure 5:**
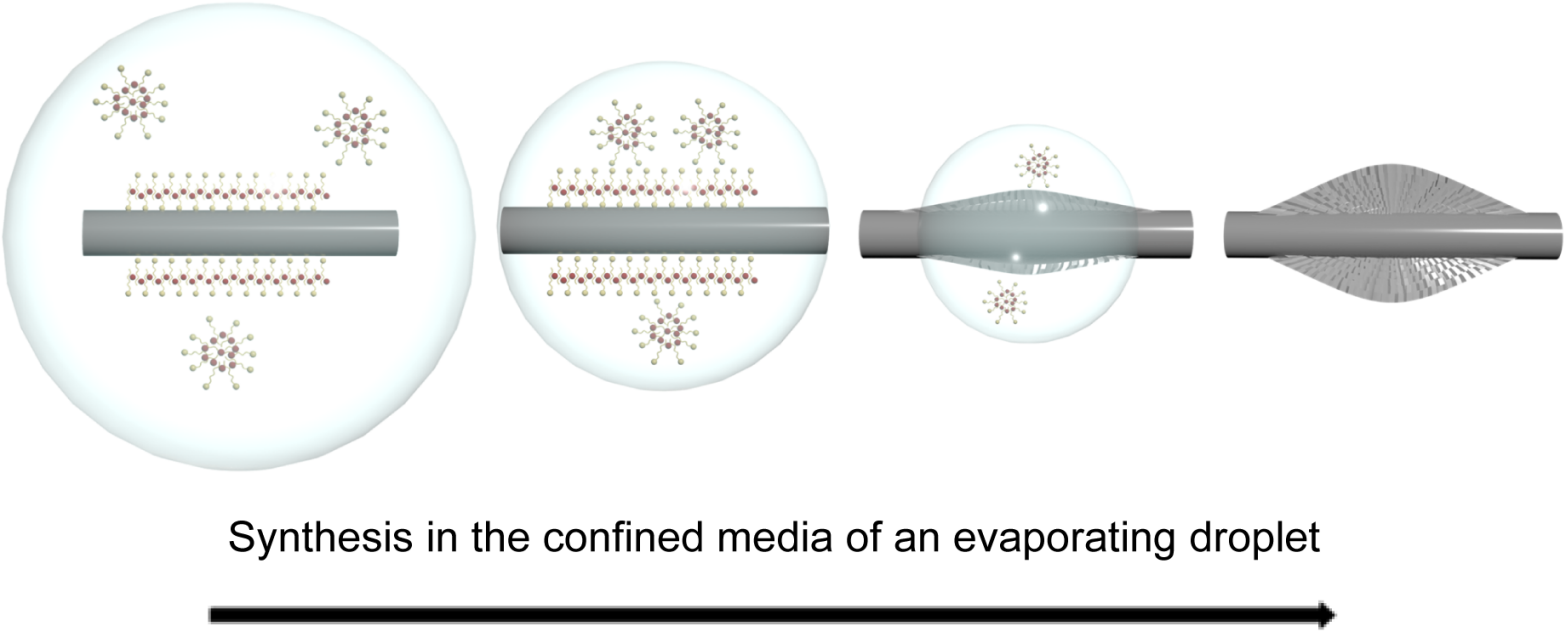
Proposed mechanism of synthesis of the MHNT composite.

Figure 6a shows the powder X-ray diffraction (XRD) patterns for MCM-41 and the MHNT composite. The diffraction peaks of MCM-41 exhibit a characteristic (100) peak at 2θ = 2.97°, corresponding to a *d*-spacing of 2.97 nm, with secondary (110) and (200) peaks at 2θ = 4.91° and 5.65°, respectively. These findings confirm the hexagonal array configuration of MCM-41.^46^ The X-ray diffraction pattern for the MHNT indicates the characteristic MCM-41 (100) peak at 2θ = 2.67°, which is shifted to the left and broadened, with the primary peak representing a *d*-spacing of 3.31 nm. The peak shift to the left and the broadening indicate larger pores and an increased pore size distribution. We attribute this observation to growth through nucleation on the HNT surface and a surface-induced strain on the MCM-41 matrix in the vicinity of the HNT surface, as illustrated by the schematic in Figure 6c.^22,34,43^

**Figure 6:**
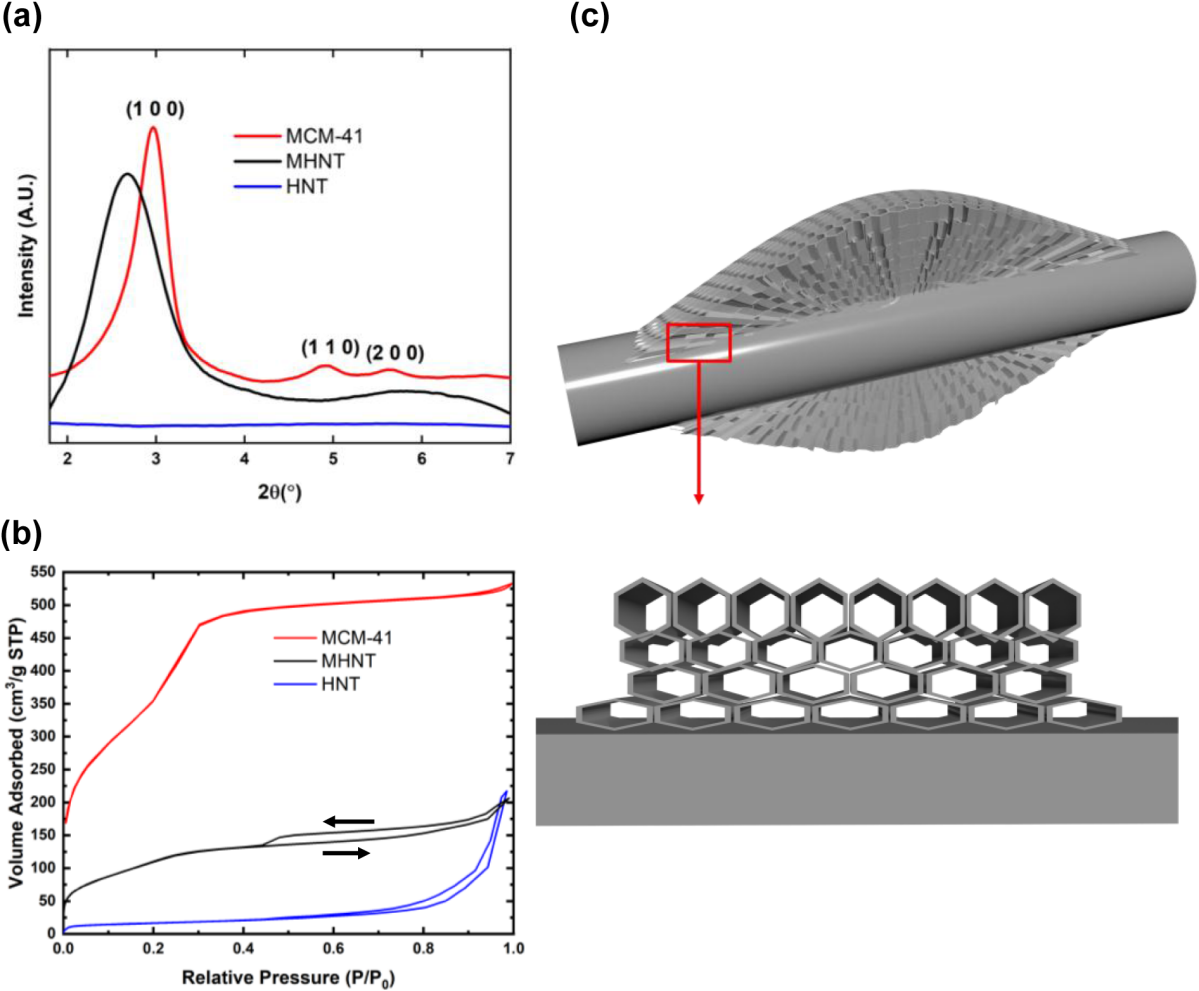
Powder X-ray diffraction analysis of (a) MCM-41 and MHNT showing the presence of the intrinsic (1 0 0) MCM-41 peak, (b) BET N_2_ adsorption isotherms for MCM-41, HNT, and MHNT, and (c) schematic of MCM-41 skin growth on the HNT surface.

The BET surface area analysis, as shown in Table 1, confirms that HNT exhibits a surface area of 57 m^2^/g, while MCM-41 displays a significantly higher surface area of 1487 m^2^/g. These values are consistent with those reported in previous studies.^22,26^ The MHNT composite shows a much higher surface area of 403 m^2^/g, which is 7 times higher than that of the HNT. This value represents a roughly weighted average derived from the MCM-41-to-HNT ratio in the composite. We also note that the formation of the MCM-41 skin on the HNT allows a significant increase in the surface area without compromising the integrity of the HNT through selective etching to increase surface area.^23,31^

**Table 1:** BET Surface Area and BJH Pore Volume of MCM-41, HNT, and MHNT

SAMPLE	BET Surface Area (m^2^/g)	BJH Pore Volume (cm^3^/g)
MCM-41	1484	0.61
HNT	57	0.34
MHNT	403	0.32

In Figure 6b, the isotherm of MCM-41 exhibits a Type IV profile with an H1 hysteresis loop, characterized by a steep increase in nitrogen uptake at relative pressures between *P*/*P*_0_ = 0.2 and 0.4. This behavior is typical of capillary condensation in cylindrical mesopores. BJH desorption analysis in this region confirms a narrow pore size distribution centered around 2–4 nm, consistent with the highly ordered mesoporous framework templated by surfactant-assisted silica assembly. The isotherm of HNT also corresponds to Type IV but with an H3 hysteresis loop, indicative of slit-like pores or hollow tubular structures with limited pore connectivity, such as the lumens of halloysite nanotubes. In contrast, MHNT displays a more complex adsorption–desorption profile. In the low-pressure region (*P*/*P*_0_ = ∼0.2–0.4), it exhibits a trend similar to that of MCM-41 but with a more gradual increase in nitrogen uptake, reflecting the partial incorporation (∼35 wt %) of MCM-41 within the composite. In the intermediate to high-pressure range (*P*/*P*_0_ = ∼0.4–1.0), MHNT shows a Type IV isotherm with an H4 hysteresis loop, which likely arises from capillary condensation within structures containing large mesopores embedded in a matrix of much smaller or partially blocked pores.^22,47–49^ Combined with the slight decrease in the total pore volume from 0.34 to 0.32 cm^3^/g, this behavior suggests that the MCM-41 network forms partially within or over some of the HNT cavities.

Fourier transform infrared (FT-IR) spectra are presented in Figure S1. These data provide evidence for the presence of amine functional groups on HNT and MHNT adsorbents. As illustrated in Figure S1, both MCM-41 and MHNT display absorption bands characteristic of silica materials.^50^ Compared with the spectrum of calcined HNT, the spectra of amine-loaded samples (30 PEI/HNT and 40 PEI/MHNT) display distinct new absorption peaks at 1566 and 1474 cm^–1^. These peaks are indicative of symmetric and asymmetric bending vibrations of the NH_2_ groups,^23,51^ respectively, confirming the presence of amine groups in the HNT and MHNT adsorbents. Additionally, the appearance of absorption bands at 2957 and 2820 cm^–1^ is attributed to the CH_2_ stretching vibrations arising from PEI in HNT and MHNT.^52^

TGA was used to quantify the amine content in both HNT and MHNT composite samples across a temperature range of 25–710 °C, with the results shown in Figure 7. The PEI (polyethylenimine)-loaded HNT samples demonstrate two distinct phases of weight loss. The initial phase, occurring from 25 to 150 °C, is associated with the expulsion of pre-absorbed water from the PEI-treated samples. The subsequent weight loss phase, from 150–460 °C, is attributed to the degradation of PEI within the HNT lumen. This analysis determined that the PEI content in the HNT adsorbents is approximately 32 wt %, closely aligning with the 30 wt % PEI used during the synthesis process. For the MHNT adsorbents, the PEI content is approximately 41 wt %, also closely aligns with the 40 wt % loading of PEI.

**Figure 7:**
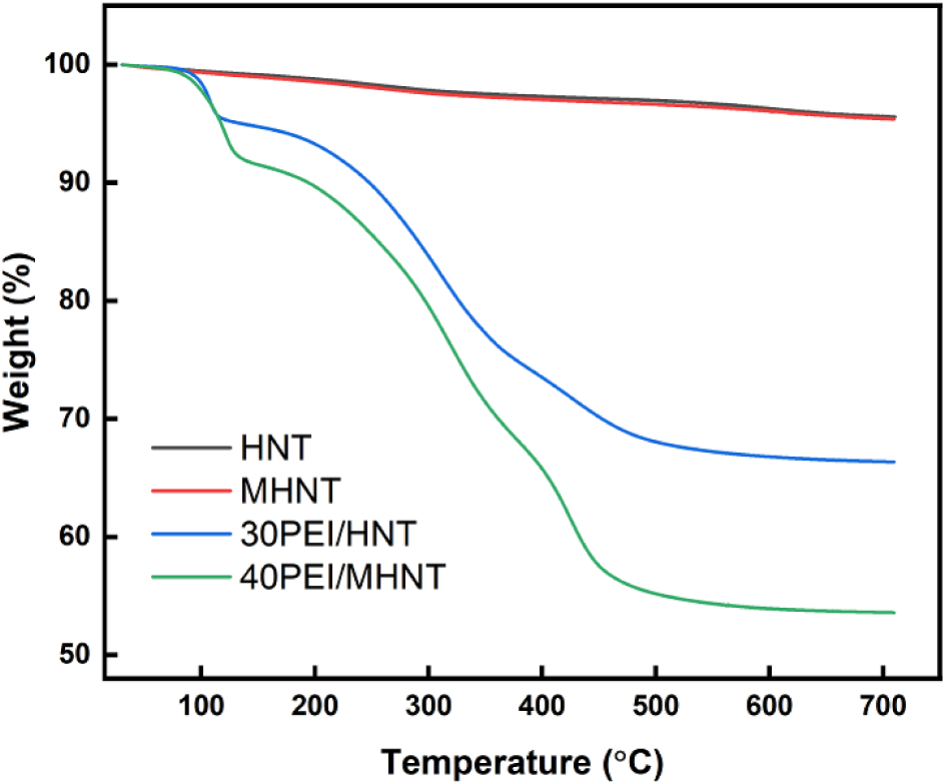
TGA of the HNT, MHNT, 30PEI/HNT, and 40PEI/MHNT samples. The TGA weight loss analysis quantitatively assesses the amine loading in HNT and MHNT, revealing the extent of PEI incorporation into these materials.

We note that the TGA characterization in Figure 7 (FTIR in Figure S1) use 30 wt % PEI in the HNT and 40 wt % PEI in the MHNT, which represent the best CO_2_ capture performance for each material, respectively. The discrepancy in PEI loadings is illustrated by the combined photographic and schematic images in Figure 8. The photographic images demonstrate the physical appearances of HNT and MHNT adsorbents, each loaded with 40 wt % PEI. Upon loading with 40 wt % PEI, the HNT sorbent exhibits an aggregated, paste-like form (Figure 8a), diverging from its initial powder form. In contrast, the MHNT composite retains a powder-like appearance even after loading with 40 wt % PEI (Figure 8b). The schematic provides insight into these observations. The paste-like form of 40PEI/HNT suggests that excess PEI forms a layer over HNT, which leads to diffusional restrictions to CO_2_ entry.^23,34^ On the other hand, the powder-like form of 40PEI/MHNT is due to PEI entry and adsorption into the mesoporous MCM-41 skin.

**Figure 8:**
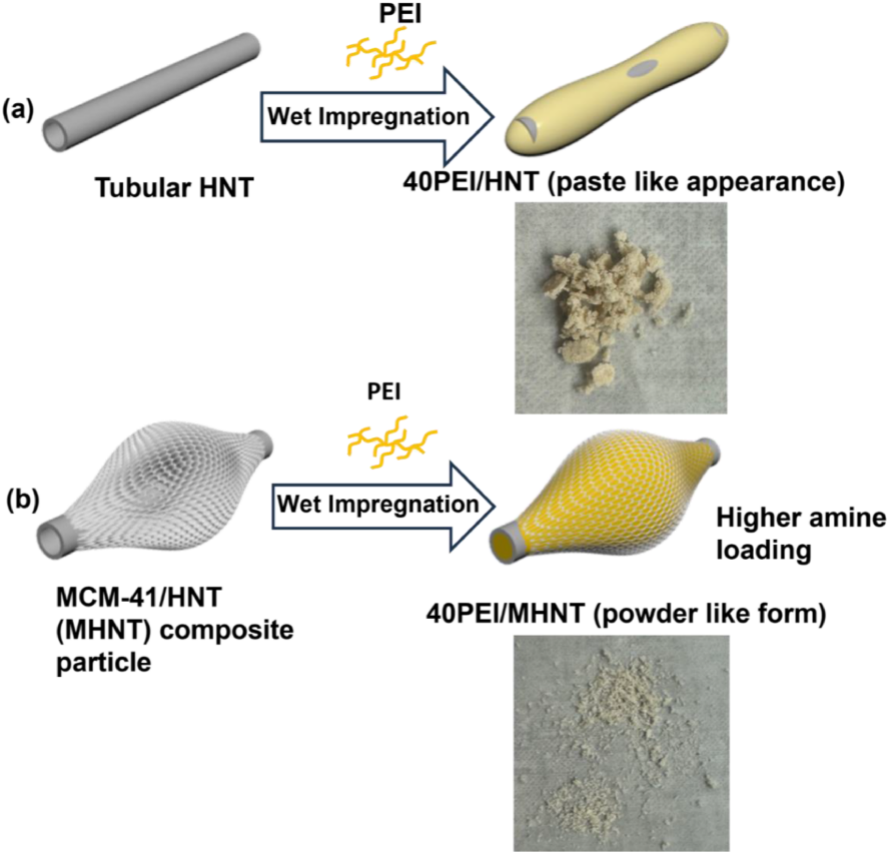
Schematic and photographic images illustrating the effects of PEI loading on HNT and MHNT adsorbents. (a) The limited surface area of the tubular HNT leads to low PEI distribution, resulting in an aggregated, paste-like form upon 40 wt % PEI loading. (b) The incorporation of a mesoporous MCM-41 skin onto HNT provides a larger surface area and pore volume, allowing for higher PEI loading while maintaining a powder-like form.

In the following section, we compare the CO_2_ capture characteristics of PEI/HNT and PEI/MHNT at various PEI loadings.

### 3.2 PEI Loading and CO_2_ Capture Analysis

Our concept for this approach involves generating a mesoporous silica skin on a clay nanotube HNT to increase its surface area. To validate this concept, we evaluated the CO_2_ capture via adsorption into PEI-loaded HNT or MHNT particles. This evaluation was conducted at 75 °C and atmospheric pressure.

In our previous study,^34^ we found that the optimal PEI loading for MCM-41 is 30 wt %, achieving a CO_2_ capture capacity of 1.16 mmol_CO2_/g_adsorbent_. The limitations of this 30 wt % PEI loading are due to the saturation of the external surface by PEI, which creates a barrier for CO_2_ diffusion and prevents further PEI penetration into the center of the MCM-41 structure.^34^ Figure 9 shows the CO_2_ adsorption capacity for PEI/HNT and PEI/MHNT at various PEI loadings from 20 to 50 wt % at 75 °C. For both adsorbents, at low PEI loadings (20 to 30 wt %), the adsorption capacities increase with increasing PEI loading. At 20 wt % loading, the halloysite nanotube (HNT) support, which has a large lumen diameter of approximately 30 nm, provides a relatively open internal structure that allows easier diffusion of CO_2_ molecules and higher accessibility to impregnated amine sites. By contrast, in the MHNT composite, although the total surface area is substantially increased due to the formation of the MCM-41 coating, the mesoporous silica layer possesses relatively small pores of about 3 nm in diameter. These smaller mesopores, while beneficial for increasing surface area and total amine loading, introduce greater diffusional resistance for CO_2_ molecules compared with the wider HNT lumen, especially at low amine loadings. Consequently, at lower PEI contents, the overall amine efficiency (defined as the amount of CO_2_ adsorbed per amount of amine) is slightly higher for PEI/HNT than for PEI/MHNT. This difference is primarily attributed to the greater ease of diffusion of CO_2_ through the larger internal lumen of the HNT, whereas in the MHNT, the tighter mesoporous structure can partially restrict access of CO_2_ to deeply impregnated amines at low loadings.

**Figure 9:**
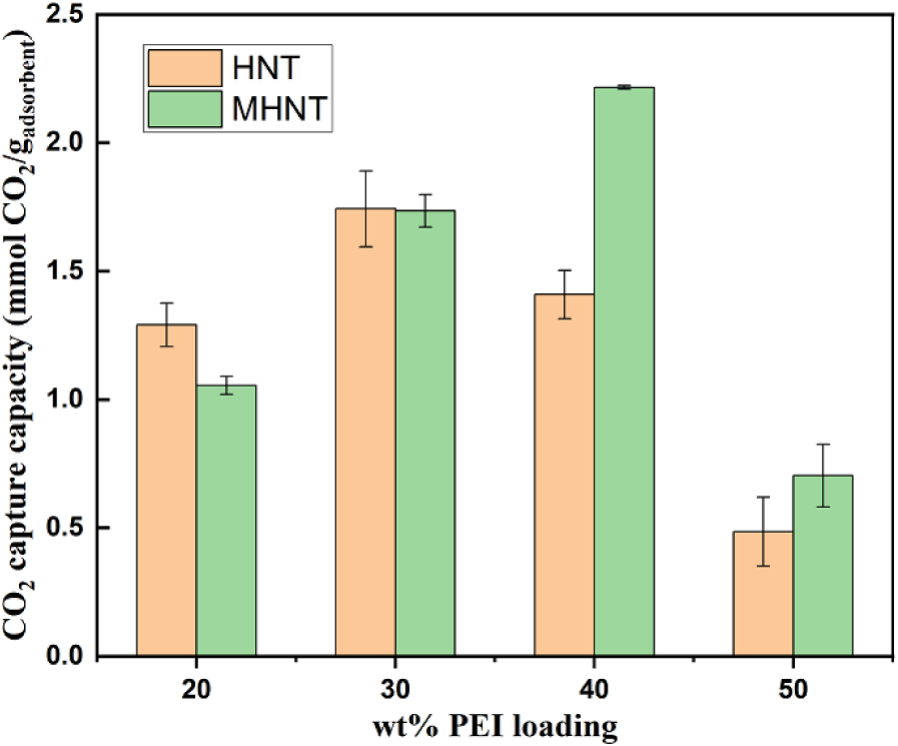
CO_2_ adsorption capacity plot showing the change in the adsorption capacity of HNT and MHNT at varying PEI loadings.

Holewinski and coworkers^53^ also noted that, for SBA-15, the amine efficiency of bulk-like polyethylenimine (PEI) is higher than that of wall-bound PEI at low to moderate loadings, as bulk-like PEI exhibits faster molecular dynamics, possibly due to greater accessibility of sorption sites compared to systems with slower PEI dynamics. In our case, at 20  wt % PEI loading, the MHNT is expected to contain a greater proportion of wall-bound PEI than the HNT, owing to its higher surface area. This structural difference likely results in a slightly lower amine efficiency for the MHNT compared to the HNT at the same PEI loading.

At 30 wt % loading, the HNT and MHNT exhibit similar CO_2_ capture capacities. With a further increase in the PEI loading to 40 wt %, the adsorption capacity of PEI/HNT decreases to 1.41 mmol_CO2_/g_adsorbent_. The MHNT adsorbent indicates an adsorption capacity of 2.22 mmol_CO2_/g_adsorbent_ at 40 wt % loading of PEI, representing a 57% increase in adsorption. Cai and coworkers^27^ also reported similar results for PEI loading in the HNT. They claimed that at 40 wt % PEI loading, polyethylenimine (PEI) filled the lumen of halloysite nanotubes (HNTs), blocking access to the internal amine groups. Additionally, excess PEI led to the agglomeration of the PEI-coated HNTs and the formation of larger solid clusters. As a result, CO_2_ molecules could only interact with a limited portion of surface-exposed PEI, while the majority of internal amine functionalities remained inaccessible. Consequently, the CO_2_ adsorption capacity decreased sharply at this high loading level. For the MHNT sample at 40 wt % PEI loading, the benefit of enhanced amine dispersion and a greater surface area in MHNT becomes dominant, yielding superior CO_2_ capture capacity compared to the HNT.

In the MHNT composite, which consists of approximately 35 wt % MCM-41 and 65 wt % HNT, a total PEI loading of 40 wt % was incorporated. Since the maximum PEI loading achievable for the pure HNT is 30 wt %, the additional 10 wt % of PEI can be attributed to the 35 wt % MCM-41 component of the composite. This corresponds to a normalized PEI loading of 52.6 wt % within the MCM-41 phase, which significantly surpasses the capacity typically attainable in bulk MCM-41. This composite architecture promotes more efficient diffusion of CO_2_ to amine-rich regions, as the thin MCM-41 layer minimizes diffusion barriers while maintaining accessibility. There is likely an optimum thickness for the mesoporous silica skin that maximizes capacity while minimizing diffusion limitations. If the MCM-41 layer is too thick, CO_2_ molecules must diffuse a longer distance through narrow channels, slowing adsorption kinetics. Furthermore, loading PEI uniformly throughout a thick shell is challenging. Conversely, if the MCM-41 coating is too thin, it may fail to provide sufficient additional porosity to meaningfully enhance amine loading, and the resulting material would be difficult to quantify or compare with the bare HNT. Therefore, careful control over the MCM-41 layer thickness is essential to balance amine accessibility, diffusion efficiency, and material characterization. In our case, the 35 wt % MCM-41 skin in the MHNT composite may not represent the best configuration, but it does enhance the CO_2_ capture efficiency.

At 50 wt % PEI loading, the CO_2_ capture capacity of HNT continues to decrease due to the same reason as at 40 wt % loading. When 50 wt % PEI is added to MHNT, the CO_2_ capture capacity decreases significantly, likely due to the support’s pores becoming saturated with the polymer; additional PEI tends to form a thick, paste-like layer on the external surface, blocking pore entrances and coating the surface of the HNTs. Farinmade and coworkers^34^ reported the use of MCM-41 impregnated with polyethylenimine (PEI) for CO_2_ capture and found that a 30 wt % PEI loading exhibited the highest CO_2_ capture capacity. However, higher PEI loadings led to reduced CO_2_ uptake. PEI loaded on the external surface itself acts as a barrier for internal PEI that was loaded in MCM-41. For our MHNT, as the high-surface-area MCM-41 is buried under excessive PEI, the composite behaves increasingly like an unmodified HNT, diminishing the advantages of the mesoporous skin. This leads to a sharp decline in the accessible surface area and pore volume.

Figure 10 shows the CO_2_ adsorption and desorption isotherms for the optimized CO_2_ capture materials, 30PEI/HNT and 40PEI/MHNT, over five cycles. Both materials exhibit excellent cyclic stability, with 40PEI/MHNT retaining 99.4% of its adsorption capacity and 30PEI/HNT retaining 99.3%. The slight decrease in CO_2_ capacity observed over repeated cycles is likely attributable to minor PEI degradation, as previously reported in the literature.^27^ These results underscore the excellent stability of both adsorbents under repeated operational conditions, highlighting their potential for long-term CO_2_ capture applications. A detailed examination of the initial uptake slopes (inset of Figure 10) reveals that both 30PEI/HNT and 40PEI/MHNT adsorbents have similar initial adsorption rates. Our hypothesis is that this similarity arises from the presence of sufficient freely available PEI to rapidly capture CO_2_ during the early stage of adsorption. In other words, the initial adsorption behavior is governed by an excess of accessible amine sites, allowing for comparable rates in both systems. Since CO_2_ adsorption is essentially an irreversible chemisorption process forming carbamate species, the initial uptake is primarily controlled by the availability of amine groups. However, the MHNT system continues to adsorb CO_2_ to a higher saturation level, as CO_2_ can also be adsorbed by the PEI that is loaded in the additional MCM-41 skin.

**Figure 10:**
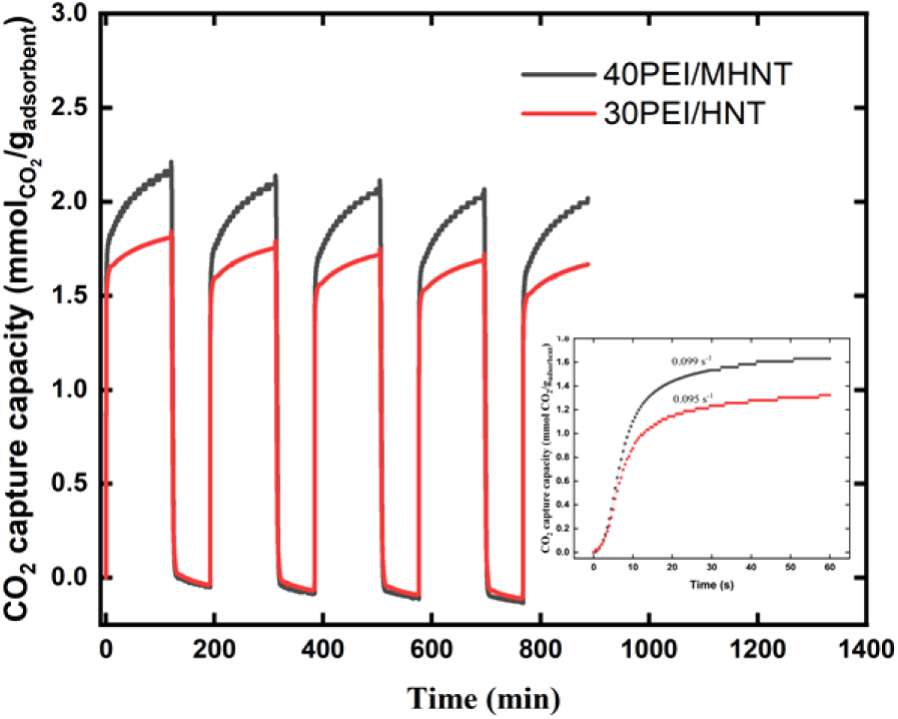
Reversibility test for CO_2_ adsorption on 30PEI/HNT and 40PEI/MHNT. The inset shows the initial uptake rate for 30PEI/HNT and 40PEI/MHNT within the first 60 s of introducing the CO_2_ gas.

## 4 Conclusions

Our study demonstrates a technique to enhance the CO_2_ adsorption capacity of polyethylenimine (PEI)-loaded halloysite nanotubes (HNTs) by generating a spindle-shaped mesoporous silica skin on the external surface of the HNT. The mesoporous silica skin of MCM-41 is synthesized using a one-step aerosol-assisted method that is facile and easily amenable to scale-up. Such mesoporous skins increase the surface area and pore volume of the composite, and result in hierarchical porosity. The spindle-shaped morphology is unique and is a consequence of synthesis in the confined environment of a rapidly evaporating aerosol droplet. We observe that the integration of the skin onto HNTs enhances the loading capacity of poly­(ethylenimine), forming a solid class I adsorbent that captures CO_2_ through chemisorption. The enhanced loading capacity of the composite leads to a 27% increase in the level of CO_2_ adsorption.

Thus, the use of mesoporous silica significantly enhances the diffusion of amine moieties into materials with large surface areas, thereby improving PEI loading and carbon capture capabilities. This method provides hierarchical porosity without compromising structural integrity, unlike alternative etching methods. These composites essentially retain the high aspect ratio of HNT and can be integrated into adsorbent beds and membranes. While the proof-of-concept example shown here simply involves encapsulation of PEI into the composite, we note that amine functionalization on the high surface area MCM-41 is also feasible, with large amine encapsulation in the lumen and small amine functionalization (e.g., aminopropyltriethoxysilane) in MCM-41. Continuing work seeks to understand the carbon capture properties of these materials under realistic flue gas conditions and potentially extrapolate these morphologies to direct air capture technologies.

The aerosol approach is versatile and adaptable to a wide range of catalytic and adsorption technologies, with significant implications for molecular transport in porous materials. The results of this study are potentially applicable to HNT-based technologies, where the tubular nature of HNTs can be used in nanoscale technologies of drug delivery and in energy and environmental applications.

## Supplementary Material



## Notes

Fourier transform infrared (FT-IR) spectroscopy analysis for HNTs, MHNTs, 30PEI/HNT, and 40PEI.MHNT (PDF)
