# Supplementary material for: Mesoporous Silica Skin on Clay Nanotubes for Carbon Capture
Source: ACS Appl Nano Mater. 2025 Jun 17;8(25):12885–94. doi: 10.1021/acsanm.5c01071 (PMC12506593; doi:10.1021/acsanm.5c01071)
Supplement: Supplementary file 1 [file an5c01071_si_001.pdf]

SUPPORTING INFORMATION

Mesoporous Silica Skins on Clay Nanotubes for Carbon Capture

Borui Wang[1], Oluwole Ajumobi[1], Jibao He[2], Julia A. Valla[3], Vijay T. John[1*]*

1. Department of Chemical & Biomolecular Engineering, Tulane University, 6823 St. Charles Avenue, New Orleans, Louisiana 70118, United States.

2. Coordinated Instrumentation Facility, Tulane University, 6823 St. Charles Avenue, New Orleans, Louisiana 70118, United States.

3. Department of Chemical & Biomolecular Engineering, University of Connecticut, Storrs, Connecticut 06269, United States.

Submitted to ACS Applied Nano Materials, June 2025.

*To whom correspondence may be addressed

Vijay T. John –vj@tulane.edu

Julia A. Valla –Ioulia.valla@uconn.edu

S-1: Fourier Transform Infrared (FT-IR) spectroscopy analysis for HNT and MHNT adsorbents

Figure S-1 shows the Fourier Transform Infrared (FT-IR) spectroscopy analysis of HNT and MHNT adsorbents. Both MCM-41 and MHNT exhibit characteristic absorption bands of silica materials.[1] In the spectra of amine-loaded samples (30PEI/HNT and 40PEI/MHNT), new absorption peaks appear at 1566 and 1474 cm^{-1}, corresponding to symmetric and asymmetric bending vibrations of the NH_2 groups respectively.[2, 3] Additional bands observed at 2957 and 2820 cm^{-1} are attributed to the CH_2 stretching vibrations arising from the presence of PEI in HNT and MHNT.[4]

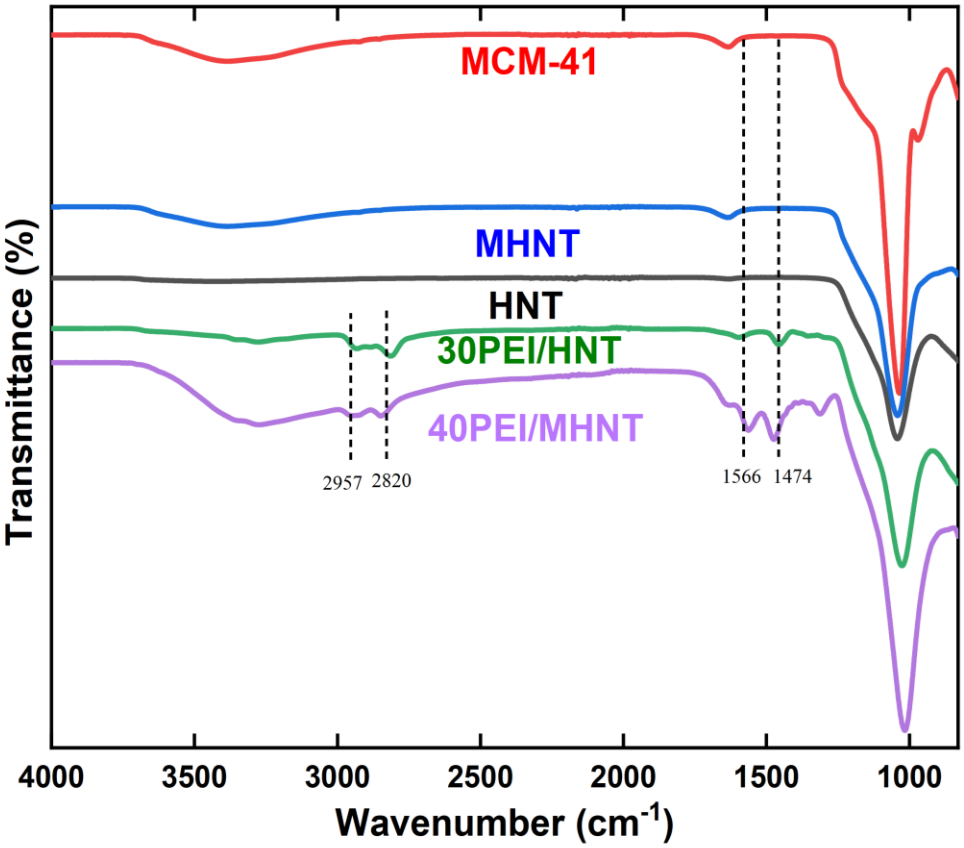

Figure S-1. Fourier transform infrared spectroscopy analysis of HNT, MHNT and PEI loaded HNT, MHNT samples. The presence of symmetric and asymmetric bending vibrations at 1566 and 1474 cm^{-1} of the NH_2 groups for PEI-loaded sample confirm the existence of amine functional groups.

REFERENCES

(1) Sarı Yılmaz, M.; Dere Özdemir, Ö.; Pişkin, S. Synthesis and characterization of MCM-41 with different methods and adsorption of Sr2+ on MCM-41. *Research on Chemical Intermediates* **2015**, *41* (1), 199-211. DOI: 10.1007/s11164-013-1182-4.
(2) Niu, M.; Yang, H.; Zhang, X.; Wang, Y.; Tang, A. Amine-Impregnated Mesoporous Silica Nanotube as an Emerging Nanocomposite for CO2 Capture. *ACS Applied Materials & Interfaces* **2016**, *8* (27), 17312-17320. DOI: 10.1021/acsami.6b05044.
(3) Liu, S.-H.; Hsiao, W.-C.; Sie, W.-H. Tetraethylenepentamine-modified mesoporous adsorbents for CO2 capture: effects of preparation methods. *Adsorption* **2012**, *18* (5), 431-437. DOI: 10.1007/s10450-012-9429-8.
(4) Tumuluri, U.; Isenberg, M.; Tan, C.-S.; Chuang, S. S. C. In Situ Infrared Study of the Effect of Amine Density on the Nature of Adsorbed CO2 on Amine-Functionalized Solid Sorbents. *Langmuir* **2014**, *30* (25), 7405-7413. DOI: 10.1021/la501284y.
